# Habituation as optimal filtering

**DOI:** 10.1016/j.isci.2024.110523

**Published:** 2024-07-16

**Authors:** Samuel J. Gershman

**Affiliations:** 1 Department of Psychology and Center for Brain Science, Kempner Institute for the Study of Natural and Artificial Intelligence, Harvard University, Cambridge, MA 02138, USA

**Keywords:** computational mathematics, Social sciences, Psychology

## Abstract

Habituation, the reduction of responding to repetitive stimuli, is often conceptualized as a kind of attentional filter, amplifying salient signals at the expense of non-salient signals. No prior account has explicitly formalized filtering principles that can explain the major characteristics of habituation. In this paper, a simple probabilistic model is developed which permits analysis of the optimal filtering problem. This model exhibits the major characteristics of habituation, while also shedding light on other, relatively neglected, characteristics. These results demonstrate that habituation can be understood as a form of optimal filtering.

## Highlights

• Habituation is the simplest form of learning, nearly universal across living organisms
• We lack a comprehensive understanding of the principles underlying habituation
• This paper proposes a model of habituation which captures its key features

## Graphical abstract

**Figure:**
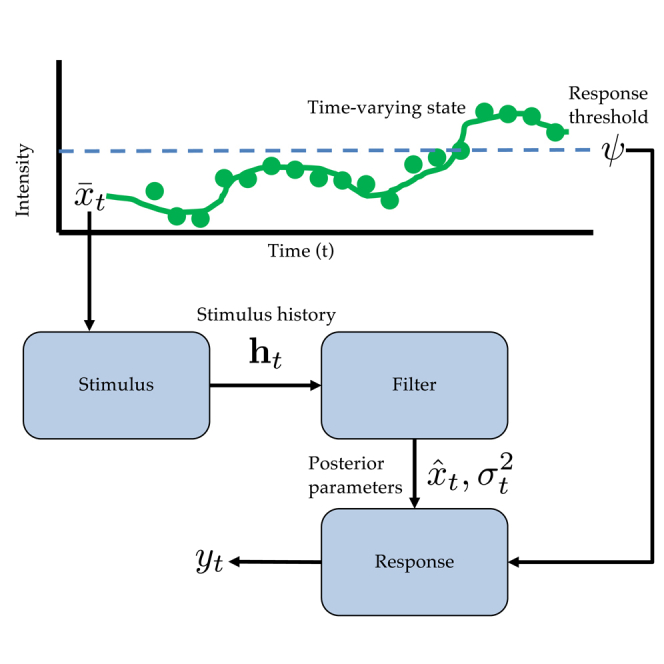

## Introduction

Arguably the simplest and most ancient form of learning is *habituation*, the reduction of responding to repetitive stimuli.^1^ Not limited to animals, it is exhibited by organisms as far flung as protozoa^2^^,^^3^^,^^4^^,^^5^ and plants.^6^^,^^7^ For example, the “sensitive plant” *Mimosa* closes its leaflets in response to mechanical stimulation, but with repeated stimulation the leaflets eventually reopen and cease to close. Similarly, the unicellular ciliate *Stentor* contracts in response to mechanical stimulation, but this contraction response attenuates with repeated stimulation. The ubiquity of habituation suggests that a universal principle may be at work. However, most theoretical treatments of habituation have focused on particular psychological^8^^,^^9^^,^^10^ or neural^11^^,^^12^^,^^13^^,^^14^^,^^15^ mechanisms, leaving the normative question—what is the logic of habituation?—unanswered.

One clue is the commonplace observation that defensive responses such as leaflet closing (in *Mimosa*) and cellular contraction (in *Stentor*) impede other activities such as photosynthesis and feeding. Thus, organisms should only respond defensively if the stimulus is really a threat. *Mimosa* reopens during rain in spite of the frequent mechanical stimulation. *Stentor*, adapted to living in turbulent ponds, returns to feeding in spite of mild disturbances to the water. Determining whether a stimulus indicates a threatening situation is fundamentally ambiguous, because the signals impinging on an organism’s sensory apparatus may be similar for threats and non-threats. This imposes an inference problem: *what’s out there*? In the language of signal processing, this corresponds to *filtering*—tracking a time-varying latent state variable based on noisy signals.^16^ The interpretation of habituation as a form of attentional filtering has a venerable history (see the review by M. Ramaswami ^15^). For now, we simply note that no previous model has formalized this idea in a way that explains all the major characteristics of habituation.

By making some assumptions about the structure of the environment and the sensors, we can derive a model of Bayes-optimal filtering. According to this model, the organism represents its state uncertainty in the form of a probability distribution, updated according to Bayes’ rule. The distribution is used to compute the probability that the state is greater than a “danger” or “salience” threshold. The model assumes that the response probability or amplitude measured experimentally corresponds to the threshold exceedance probability. This simple model can explain the major characteristics of habituation (Box 1), as well as some subtle characteristics that have received less attention.

Box 1 **Major characteristics of habituation (adapted from 17)**

1. Simple habituation: repeated application of a stimulus results in a progressive decrease in the response until an asymptotic level is reached.
2. Spontaneous recovery ^18^^,^^19^^,^^20^^,^^21^: if the stimulus is withheld after response decrement, the response recovers at least partially over the observation time.
3. Potentiation ^22^^,^^19^^,^^4^: after multiple series of stimulus repetitions and spontaneous recoveries, the response decrement becomes successively more rapid and/or more pronounced.
4. Frequency (rate) sensitivity ^23^^,^^24^^,^^21^: other things being equal, more frequent stimulation results in faster and/or more pronounced response decrement, and more rapid spontaneous recovery.
5. Intensity sensitivity ^19^^,^^25^^,^^23^: within a stimulus modality, the less intense the stimulus, the more rapid and/or more pronounced the behavioral response decrement. Very intense stimuli may yield no significant observable response decrement.
6. Stimulus specificity ^26^^,^^27^^,^^28^: within the same stimulus modality, the response decrement shows some stimulus specificity. This characteristic distinguishes habituation from sensory adaptation/motor fatigue in neuroscience.
7. Dishabituation ^29^^,^^19^: presentation of another (usually strong) stimulus results in the recovery of the habituated response.
8. Habituation of dishabituation ^30^^,^^31^^,^^32^^,^^33^^,^^19^: upon repeated application of the dishabituating stimulus, the amount of dishabituation produced decreases.
9. “Below-zero” effects ^19^: the effects of repeated stimulation may continue to accumulate even after the response has reached an asymptotic level. This effect of stimulation beyond asymptotic levels can alter subsequent behavior, for example, by delaying the onset of spontaneous recovery.
10. Long-term effects ^34^^,^^35^: some stimulus repetition protocols may result in properties of the response decrement (e.g., more rapid rehabituation than baseline, smaller initial responses than baseline, smaller mean responses than baseline, less frequent responses than baseline) that last hours, days or weeks. We do not explicitly model these effects here, since the model does not commit to any particular timescale.

## Results

### Model

**Figure 1:**
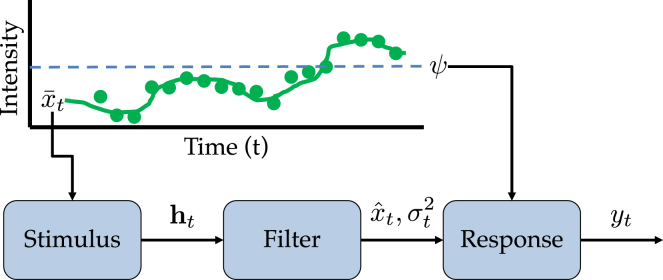
Structure of the model A stimulus generates a sensory signal with intensity $x_{t}$ (green circles), drawn from a Gaussian distribution with mean intensity ${\bar{x}}_{t}$ (green line). All the sensory signals up to time *t* are collected into the vector $h_{t}$, which is fed into the learning system (a Bayes-optimal filter). The output of the learning system is a probabilistic estimate of the mean intensity at time *t*, parametrized by a mean (${\hat{x}}_{t}$) and variance ($\sigma_{t}^{2}$). Based on this estimate relative to a threshold ψ (dashed blue line), the organism generates a binary response with probability $y_{t}$.

We model an organism living in a time-varying environment (Figure 1). At time *t*, the organism collects sensory signal $x_{t}\in R$, drawn from a probability distribution $p\left(x_{t}|{\bar{x}}_{t}\right)$ with expectation $E\left[x_{t}\right]={\bar{x}}_{t}$. We will refer to ${\bar{x}}_{t}$ as the *state* at time *t*. The state’s time series is drawn from a probability distribution $p\left(\bar{x}\right)$. The sensory signal is assumed to be measured on a logarithmic scale, consistent with psychophysical (i.e., the Weber-Fechner law^36^) and biophysical (i.e., fold-change detection^37^^,^^38^) principles. The model developed below does not intrinsically require logarithmic sensory transduction, but this is mathematically convenient since it does assume that the signals are real-valued.

The organism generates a response $y_{t}\in \left[0,1\right]$ based on its inferences about the underlying state. In particular, we assume that the response corresponds to the probability that the state is greater than a threshold ψ:

(Equation 1) $$y_{t}=p\left({\bar{x}}_{t}>\psi |h_{t}\right)=\int_{{\bar{x}}_{t}}I\left[{\bar{x}}_{t}>\psi \right]p\left({\bar{x}}_{t}|h_{t}\right)d{\bar{x}}_{t}\text{,}$$

where I[·]=1 if its argument is true (0 otherwise), and $p\left({\bar{x}}_{t}|h_{t}\right)$ is the posterior over the state conditional on the signal history $h_{t}=\left\{x_{t^{\prime }}:t^{\prime }\leq t\right\}$, given by Bayes’ rule:

(Equation 2) $$p\left({\bar{x}}_{t}|h_{t}\right)\propto p\left(h_{t}|{\bar{x}}_{t}\right)p\left({\bar{x}}_{t}\right)\text{.}$$

Depending on the preparation under study, we can interpret the response $y_{t}$ either as the probability of a binary action (e.g., the probability of contraction in response to an aversive mechanosensory signal) or as the amplitude of a continuous action (e.g., the amplitude of the flexor withdrawal in response to cutaneous electrical stimulation).

To obtain an analytically tractable model, we assume that the state is drawn from a Gaussian process^39^ and then corrupted by additive Gaussian noise to generate the signal:

(Equation 3) $$\bar{x}∼\text{GP}\left(m,k\right)$$

(Equation 4) $$x_{t}∼N\left({\bar{x}}_{t},\alpha \right)\text{,}$$

where $m_{t}=E\left[{\bar{x}}_{t}\right]$ is the mean function, $k_{t,t^{\prime }}=E\left[\left({\bar{x}}_{t}-m_{t}\right)\left({\bar{x}}_{t^{\prime }}-m_{t^{\prime }}\right)\right]$ is the covariance function, and α>0 is the signal noise variance. We assume that the mean function is fixed to 0 for all *t*, which means that the organism tends to expect 0 signal amplitude in the absence of evidence to the contrary. The covariance function determines the timescale of habituation; we do not explicitly distinguish between “short-term” and “long-term” habituation (see Property 10 in Box 1) because the meaning of these terms (how long is long?) vary depend on the context and model organism.

**Figure 2:**
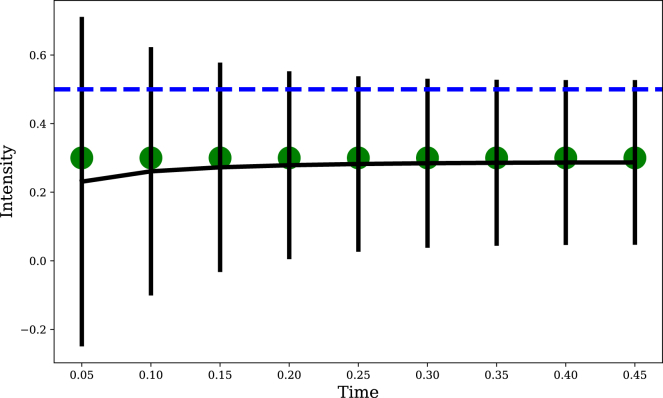
Illustration of the model Green dots show the stimulus series and blue dashed line shows the response threshold (as in Figure 1). The black curve shows the posterior mean (with standard deviation error bars) just prior to the signal at each time point. In this illustration, the mean doesn’t change very much over time, while the standard deviation shrinks gradually. This has the effect of decreasing the probability $y_{t}$ that the signal mean ${\bar{x}}_{t}$ is above the threshold ψ, thus producing habituation.

Under these assumptions, we can derive a closed-form expression for Equation 1:

(Equation 5) $$y_{t}=\Phi \left(\frac{{\hat{x}}_{t}-\psi }{\sigma_{t}}\right)\text{,}$$

with posterior mean and variance given by:

(Equation 6) $${\hat{x}}_{t}=k_{t}^{⊤}{\left(K+\alpha I\right)}^{-1}h_{t}$$

(Equation 7) $$\sigma_{t}^{2}=k_{t,t}-k_{t}^{⊤}{\left(K+\alpha I\right)}^{-1}k_{t}\text{,}$$

where $k_{t}$ is the vector of covariances between *t* and all other time points, K is the matrix of covariances evaluated at all time points, and the signal history $h_{t}$ is organized into a column vector. Note that the expressions for the posterior mean and variance hold for any choice of covariance function (including non-stationary and non-smooth ones). Figure 2 illustrates how the model works on an example habituation protocol.

For simplicity, the model above was presented for the single stimulus case. It can be straightforwardly generalized to settings with multiple stimuli by assuming that the covariance function is defined over time-stimulus pairs z=[t,s], where $s\in R^{D}$ denotes a *D*-dimensional stimulus.

Further modeling details can be found in the STAR methods section.

### Simulations

Let us begin with the basic phenomenon of habituation (Property 1 in Box 1): why does it happen? The filtering model asserts that habituation arises from the process of learning that the current state is non-threatening. Intuitively, a repeated signal, provided that its intensity is below the threshold ψ, will drive the mean ${\hat{x}}_{t}$ of the posterior distribution toward a sub-threshold value. In addition, the variance of the posterior distribution $\sigma_{t}^{2}$ will be driven toward 0 as evidence accumulates. Together, these dynamics imply that the response $y_{t}$ should diminish with repetition—i.e., simple habituation.

**Figure 3:**
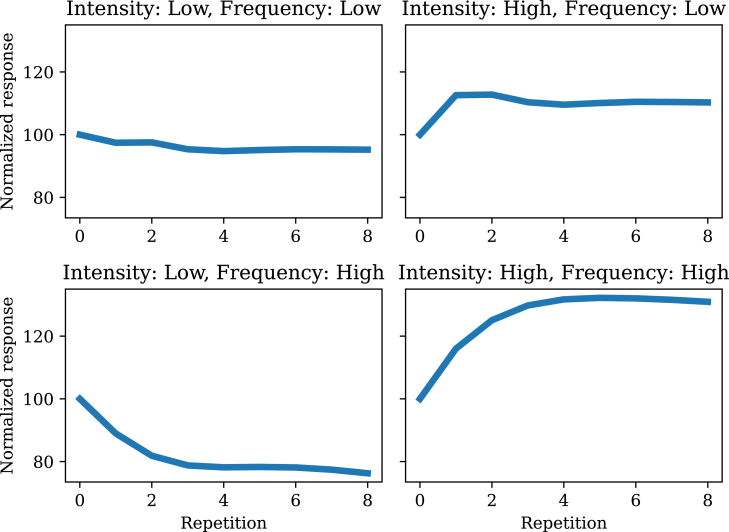
Stimulus frequency and intensity effects When intensity is low, higher frequency stimulus presentations lead to stronger habituation. When intensity is high, habituation gives way to sensitization (increased responding as a function of stimulus repetition), with stronger sensitization for high frequency stimulus presentations.

Parametric studies of stimulus frequency (or rate) and intensity have revealed a more complex pattern. A common finding (e.g., ^21^^,^^24^) is that habituation is stronger for high stimulus frequency. The filtering model accounts for this (Figure 3) by virtue of the fact that high frequency drives the posterior uncertainty down more aggressively, yielding a sharper reduction in responding. Critically, however, this depends on the intensity being below threshold. If it is above threshold, *sensitization* (an *increase* in responding with repetition) should be observed. This is consistent with the findings of Groves and Thompson,^23^ who further demonstrated that high frequency stimuli produce stronger sensitization. In other words, frequency controls the slope of the learning curve, which goes in opposite directions depending on stimulus intensity. This is expected based on the different roles for the posterior mean and variance in determining response dynamics. Specifically, intensities below the response threshold will produce habituation, whereas intensities above the response threshold will produce sensitization. Because high frequency reduces the variance more quickly, this translates to a faster learning curve.

**Figure 4:**
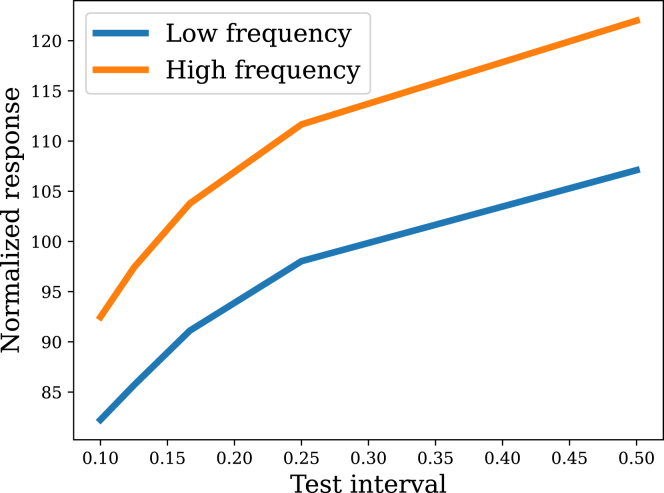
Common test procedure In an initial habituation series (not shown here), the model received either low or high frequency stimulation. It was subsequently tested on multiple intermediate intervals in random order.

Most studies that manipulate parametric properties of the stimulus series confound learning (encoding and retention of information) and performance effects (expression of learned knowledge); see the review by R.M.Colwill et al.^40^ for further discussion of this issue. A few studies have used a “common test” procedure, where the learning conditions are varied across groups of subjects but all subjects receive the same test trials. Here, we focus on a study reported by Davis,^24^ who showed that responses to test stimuli were weaker for low frequency stimuli when measured using a common test. The filtering model captures this pattern (Figure 4).

This observation poses an apparent paradox, since the studies reviewed previously (which did not use the common test procedure) showed stronger habituation (i.e., weaker responses) for high frequency stimuli. The paradox can be resolved by recognizing that responding recovers more quickly for high frequency compared to low frequency stimuli. In terms of the model dynamics, the same reduction in posterior uncertainty that drives amplification of sensitivity to changes in the posterior mean also drives faster updating (similar to the effects of uncertainty on learning in other domains; e.g., ^41^^,^^42^^,^^43^^,^^44^). These two roles can work against each other, thus explaining some of the non-monotonicities in the learning curves shown in Figure 3. Which role dominates will depend on both the parameters of the model and the structure of the stimulus series.

**Figure 5:**
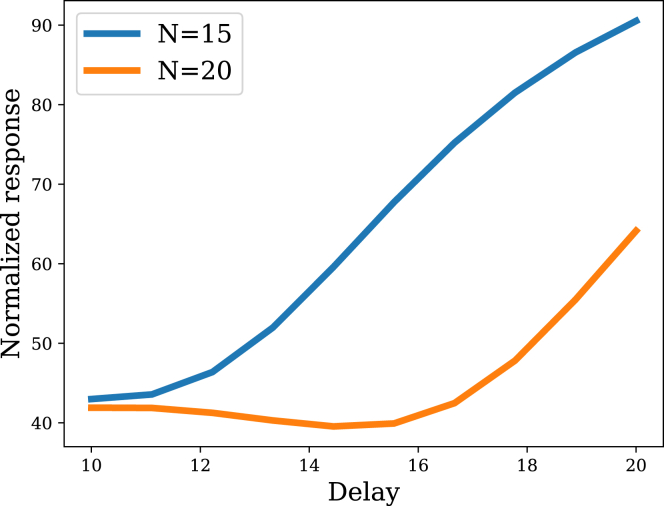
Spontaneous recovery After a rest period in which no stimuli are presented, the response recovers from habituation. Recovery is slower when the initial stimulus series was longer. Note that this plot shows single tests at different delays, not repeated tests.

Our discussion of the common test procedure highlights the fact that habituated responses recover spontaneously after a rest period.^18^^,^^19^^,^^20^^,^^21^ Recovery is slower following extended habituation, even after responding has gone to zero or some asymptotic level (so-called “below-zero” habituation; though see the studies by LE Gardner and D. Stephenson et al.^45^^,^^46^ for divergent results). The filtering model captures this phenomenon (Figure 5): extended habituation, even after asymptotic responding, further decreases the posterior variance, such that it subsequently requires more time to return to baseline.

**Figure 6:**
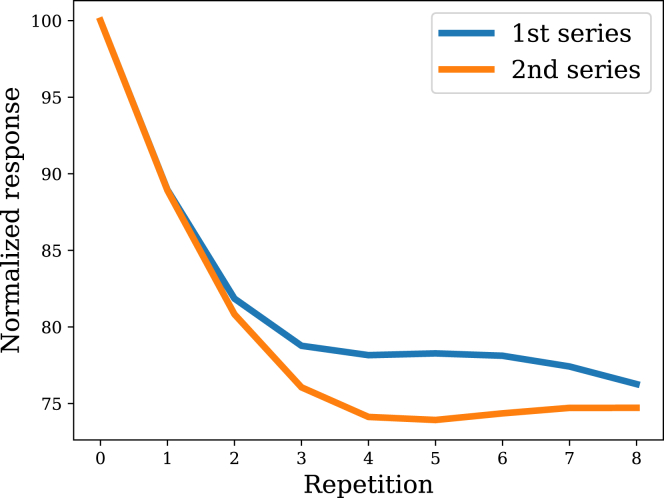
Potentiation Rehabituation to a 2nd series is faster than initial habituation to the 1st series.

Rehabituation with a 2nd series of stimulus repetitions produces a learning curve that decreases more quickly than habituation to the 1st series, a phenomenon known as *potentiation*.^4^^,^^19^^,^^22^ Importantly, the learning curve starts at roughly the same response level (provided there is adequate recovery time). This implies that potentiation is not simply a reflection of residual habituation from the 1st series—an “inactive memory”^47^ must be present, encoding information about the stimulus history that is not immediately expressed in behavior. Figure 6 shows that the filtering model captures potentiation. The response returns to baseline after the recovery period, but the posterior variance is lower (due to having more data compared to the beginning of the 1st series), which sharpens the sensitivity of the response function to changes in the posterior mean.

**Figure 7:**
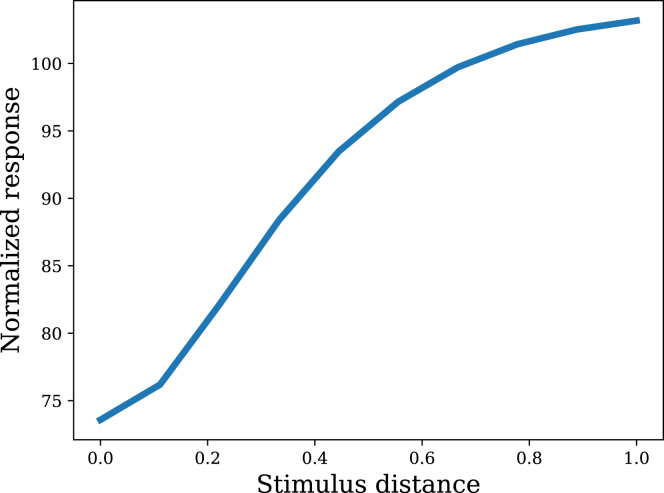
Stimulus specificity Presentation of a novel stimulus produces a generalization decrement of habituation.

So far we have focused on studies with a single stimulus. We now turn to studies with multiple stimuli. The most elementary observation is that habituation exhibits stimulus specificity: responding increases when tested on another stimulus.^26^^,^^27^^,^^28^ This has sometimes been interpreted to reflect generalization,^19^^,^^48^ in the sense that responding is intermediate between baseline and the habituated response to the familiar stimulus. Figure 7 shows that the filtering model captures graded stimulus specificity as a function of distance between the familiar and novel stimulus. This result is essentially baked into the structure of the covariance function (see STAR methods), which assumes that covariance drops off exponentially as a function of Euclidean distance in stimulus space. In other words, presenting a new stimulus moves the recent stimulus history into a new part of the stimulus space, producing a generalization decrement.

**Figure 8:**
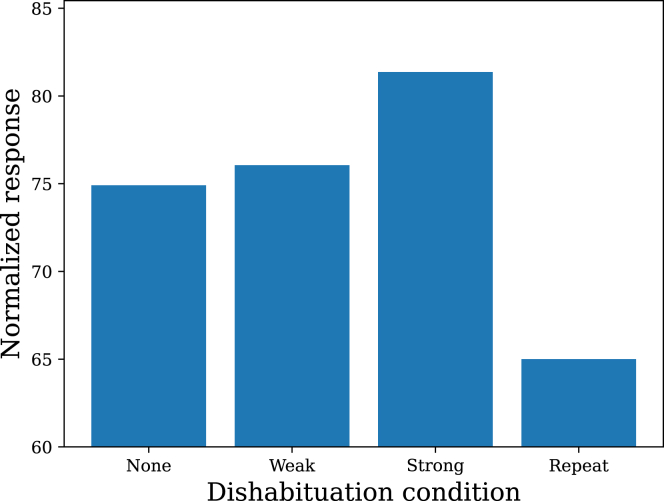
Dishabituation Presentation of a novel stimulus increases responding to the familiar stimulus. This is particularly pronounced for high intensity (strong) compared to low intensity (weak) novel stimuli. Repetition of the novel stimulus causes habituation of dishabituation.

Another important multiple-stimulus phenomenon is *dishabituation*, the increase in responding to the familiar stimulus after presentation of a novel stimulus.^19^^,^^29^ A typical (though not universal) finding is that dishabituation is stronger with high intensity novel stimuli. This arises in the model (Figure 8) because a strong stimulus is farther away in stimulus space from the familiar stimulus; the posterior probabilities of the two stimuli are coupled together, thus pulling the posterior mean for the familiar stimulus away from where it was at the end of habituation. Dishabituation itself habituates over the repeated presentations of the novel stimulus.^19^^,^^30^^,^^31^^,^^32^^,^^33^ This is also predicted by the model due to the reduction of uncertainty in the new part of stimulus space, which mitigates the generalization decrement due to moving the recent stimulus history away from the habituated part of stimulus space.

An interesting prediction of this theory is that stimulus specificity and dishabituation should be positively correlated: dishabituation should be stronger to the extent that the original habituation is stimulus-specific. This prediction follows from the fact that both stimulus specificity and dishabituation arise in the model due to the same underlying property, namely generalization decrement due to a change in the posterior mean.

### Alternative parametric assumptions

The flexibility of the Gaussian process model allows us to explore other modeling assumptions. We assumed that the covariance function is smooth over a characteristic timescale (see STAR methods). Alternatively, we could assume that the process is very non-smooth over this timescale (close to independent samples across time points), or even smoother (close to a constant mean across time points). What are the consequences of these alternative modeling assumptions?

**Figure 9:**
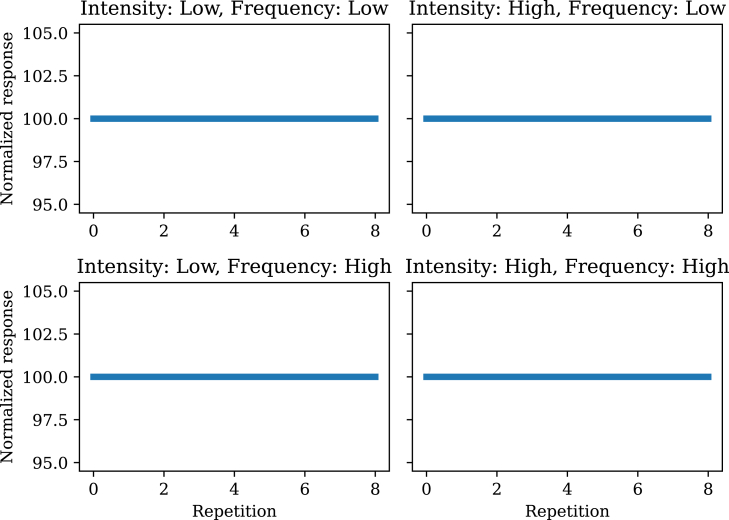
Stimulus frequency and intensity effects with a short length-scale Compare with Figure 3.

**Figure 10:**
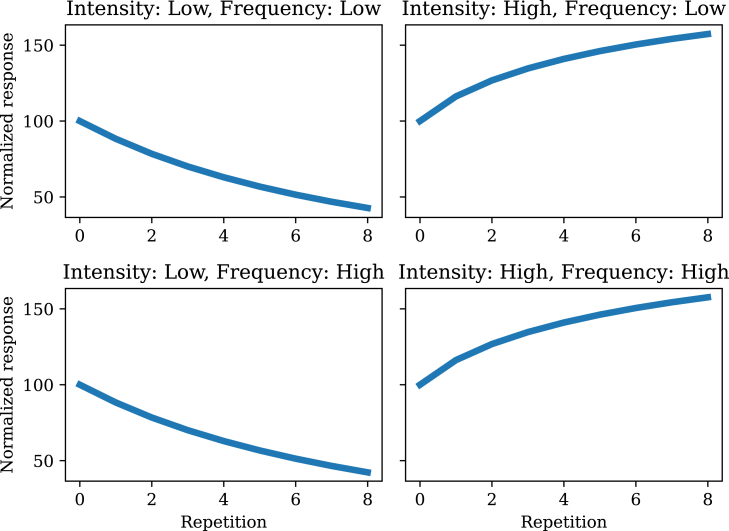
Stimulus frequency and intensity effects with a long length-scale Compare with Figure 3.

We will use the frequency and intensity simulations as a case study (compare with Figure 3). When the covariance function is very non-smooth (length-scale parameter λ=0.001), habituation and sensitization go away completely (Figure 9). This happens because there is no generalization across time points. When the covariance function is close to constant (length-scale parameter λ=100), habituation for low intensity and sensitization for high intensity is still observed, but frequency dependence goes away (Figure 10). This happens because changes in frequency around the characteristic timescale of the experiment are effectively invisible to the model, which can only detect changes at much slower time-scales.

In summary, capturing the key phenomena of habituation requires a choice of covariance function that is smooth at the characteristic timescale of the experimental protocol.

## Discussion

This paper has shown that the major characteristics of habituation can be accounted for by a model of habituation as optimal filtering. The key computations are: (i) tracking the posterior probability of a hidden state over time using Bayesian inference; (ii) mapping the posterior distribution to a response by computing the probability that the state exceeds a “threat” or “salience” threshold. These computations allow the organism to filter out unimportant signals and amplify important ones.

Related ideas have a long history in the study of habituation. Sokolov^49^ was perhaps the first to suggest that habituation reflects a form of internal model, which he conceptualized as stimulus expectation (though he never formalized this idea). Response strength depends on a comparison between the stimulus and the expectation. In other words, response strength is proportional to prediction error. Variations of this “comparator” model were later explored by Konorski^50^ and Wagner,^51^ framed in terms of comparison with memory representations (“gnostic units”) associatively activated by the stimulus history. Comparator theory resonates with modern computational ideas about predictive coding.^15^ However, with the exception of Wagner’s SOP model and Staddon’s models (discussed further below), few versions of comparator theory have been described in sufficient formal detail (and implemented in computational models) to be evaluated systematically. The adequacy of Wagner’s model as a descriptive account has been questioned by Mackintosh.^52^ For example, Wagner’s model posits separate short-term and long-term habituation processes to explain phenomena like the frequency sensitivity, but Mackintosh points out that it could be explained by a single habituation process; indeed, the filtering model only appeals to a single process.

The filtering model shares with comparator models the idea that habituation reflects interpretation of signals through an internal model. It also hypothesizes a comparison operation, though not between the stimulus and the model expectation. Instead, the comparison is between an inferred state and a threshold. In this respect, it bears similarity to “optimal approach” models developed in behavioral ecology,^53^^,^^54^ where the critical computation for organisms is the decision about whether an unfamiliar object is safe to approach.

Within the human developmental literature, normative models of habituation have been used to explain a quite different set of empirical phenomena (mainly infant looking time data). These models also invoke statistical inference, linking habituation to optimal information acquisition.^55^^,^^56^^,^^57^

None of these prior models have been comprehensively applied to all the major characteristics of habituation, as defined in Box 1. The most systematic attempt is the recent paper by Uribe-Bahamonde et al.,^10^ which could explain many aspects of habituation but nonetheless did not offer an account of several phenomena addressed here (sensitization, frequency dependence of rehabituation). Other prominent models of habituation, such as the cascaded integrator models explored by Staddon et al.^8^^,^^9^ and Bonzanni et al.,^58^ also only explain a subset of the relevant data. For example, Staddon’s models do an excellent job explaining the frequency dependence of habituation, but they do not account for sensitization. They also only apply to single stimuli, and hence cannot explain stimulus specificity or dishabituation.

Our goal is not to pit the filtering model against these other models, because they are conceptualized at different levels of analysis. Unlike these other models, the filtering model is deliberately agnostic about psychological and neural mechanisms; it adopts a level of abstraction that allows us to recognize the *function* of habituation. In principle, a number of the mechanistic ideas formalized by other models could be used to implement the computations required by optimal filtering. For example, it is possible to cast many Gaussian processes, either exactly or approximately, in a “state space” form governed by linear-Gaussian dynamics.^59^ The optimal filtering solution is then given by a Kalman filter, which is essentially a kind of leaky integrator model similar to those studied by Staddon and colleagues. Much work remains to be done in bridging these levels of analysis.

Another way in which there is convergence across levels of abstraction concerns the use of memory, which plays a prominent role in Wagner’s SOP model and Staddon’s cascaded integrator models. These models assume that a memory trace of stimulus history is maintained over time and compared with incoming stimuli. The filtering model also maintains a memory trace of the stimulus history ($h_{t}$), but this is an infinite-capacity idealization which we don’t assume is physically encoded. Because the covariance function determines the characteristic timescale over which memory needs to be maintained, very old memories can be safely discarded. One way to view the filtering model is that it stipulates what memory needs to be stored, given some assumptions about the environment. This provides a normative framework for constraining mechanistic models of habituation.

Adopting a high level of abstraction is important for understanding habituation, because evidence suggests that there may not be a single mechanism underlying all its manifestations. Studies of organisms with very simple nervous systems like *Aplysia* have suggested that homosynaptic plasticity is the mechanism of habituation,^60^ but this hypothesis does not generalize to organisms with more complex nervous systems, where multi-cellular circuit mechanisms come into play.^15^^,^^61^ Even for *Aplysia*, the mechanisms for habituation appear to be much more complex than originally envisioned.^62^ At the other extreme, unicellular organisms such as ciliates and ameobae exhibit habituation despite having no nervous system at all. Plants and even isolated cell lines^63^ are in the same boat. Models like the one developed here may help us understand what computational principles these radically different systems have in common.

### Limitations of the study

The downside of abstraction is that the model does not have much to say about the specific biological mechanisms underlying habituation in particular systems. For example, why does habituation appear to be a multicellular phenomenon in some systems but not others? Future work will need to ground the abstract model in particular biological implementations.

Another limitation is that the model assumes that the underlying latent variable generating sensory signals varies smoothly in time. However, other temporal structures might be more natural in certain environments. For example, some environments contain periodic or non-stationary temporal structures. Fortunately, these kinds of structures can be easily accommodated by the model through different choices of covariance function. We leave an exploration of this question to future work.

## STAR★Methods

### Key resources table

**Table:** 

REAGENT or RESOURCE	SOURCE	IDENTIFIER
**Software and algorithms**		

Simulation code for reproducing all the figures	The author	https://github.com/sjgershm/habituation

### Resource availability

#### Lead contact

Samuel Gershman, gershman@fas.harvard.edu.

#### Materials availability

No materials were generated as part of this study.

#### Data and code availability

• No data were generated or analyzed as part of this study.
• Simulation code for reproducing all the figures is available at https://github.com/sjgershm/habituation.
• Any additional information required to run the simulations reported in this paper is available from the lead contact upon request.

### Method details

#### Covariance function

While there is a wide range of possible covariance functions, we assume stationarity (i.e., the covariance structure depends only on $t-t^{\prime }$) and smoothness (i.e., at least first-order differentiability). A conventional choice of covariance function satisfying these assumptions is the squared exponential:

(Equation 8) $$k_{t,t^{\prime }}=\exp\;\left(-\frac{{\left|t-t^{\prime }\right|}^{2}}{2\lambda^{2}}\right)\text{,}$$

where λ>0 is the *length-scale*, which determines characteristic timescale over which fluctuations tend to occur. In the multiple stimulus case with z=[t,s], the squared exponential covariance function becomes:

(Equation 9) $$k_{z,z^{\prime }}=\exp\;\left(-\frac{{\left\|z-z^{\prime }\right\|}^{2}}{2\lambda^{2}}\right)$$

In the simulations reported above, we assume that the stimulus space is 1-dimensional; this assumption is sufficient to capture the relevant empirical phenomena. However, more generally it is plausible that the stimulus space is multi-dimensional.

#### Model parameters

All simulations are based on a fixed set of parameters: α=0.3,λ=1,ψ=0.5. In principle, the parameters could be adapted based on the stimulus history. However, this is unnecessary for the key results, so we leave the topic of parameter adaptation to future work.

#### Response normalization

Following a standard normalization procedure in the analysis of animal habituation data,^19^ model responses were multiplied by 100 and divided by the response to an isolated stimulus. This allows us to interpret a normalized response of 100 as an unhabituated reference point.
